# Real pandemic world results of pharmacokinetic-tailored personalized prophylaxis of bleeds in Polish children and adolescents with severe hemophilia A

**DOI:** 10.3389/fped.2023.1084539

**Published:** 2023-02-23

**Authors:** Tomasz Urasiński, Klaudia Paczóska, Wanda Badowska, Halina Bobrowska, Łucja Dakowicz, Grzegorz Dobaczewski, Elżbieta Latos-Grażyńska, Grażyna Karolczyk, Anna Klukowska, Andrzej Kołtan, Magdalena Wojdalska, Paweł Łaguna, Maciej Niedźwiedzki, Danuta Pietrys, Julia Radoń-Proskura, Monika Radwańska, Iwona Rurańska, Tomasz Szczepański, Dariusz Wasiński, Irena Woźnica-Karczmarz, Karolina Zielezińska, Aleksandra Królak, Tomasz Ociepa

**Affiliations:** ^1^ Department of Pediatrics, Hemato-Oncology and Gastroenterology, Pomeranian Medical University, Szczecin, Poland; ^2^ Department of Pediatric Oncology and Hematology, School of Medicine, University of Warmia Mazury, Olsztyn, Poland; ^3^ Wielkopolska Child Health Center, Poznań, Poland; ^4^ Department of Pediatric Oncology and Hematology, Medical University of Białystok, Białystok, Poland; ^5^ Department of Bone Marrow Transplantation, Pediatric Oncology and Hematology, Wrocław Medical University, Wrocław, Poland; ^6^ II Department of Pediatrics, Collegium Medicum, Jan Kochanowski University, Kielce, Poland; ^7^ Department of Pediatric Oncology, Hematology and Transplantation, Medical University of Warsaw, Warszawa, Poland; ^8^ Department of Pediatrics, Hematology and Oncology, Collegium Medicum, Nicolaus Copernicus University, Bydgoszcz, Poland; ^9^ Department of Pediatrics, Oncology and Hematology, Medical University of Lodz, Łódź, Poland; ^10^ Department of Pediatrics, Hematology and Oncology, Medical University of Gdańsk, Gdańsk, Poland; ^11^ Department of Pediatric Oncology and Hematology, Collegium Medicum, Jagiellonian University, Kraków, Poland; ^12^ Department of Pediatric Oncology and Hematology, College of Medical Sciences, University of Rzeszów, Rzeszów, Poland; ^13^ Department of Pediatrics, Hematology and Oncology, Medical University of Silesia, Katowice, Poland; ^14^ Department of Pediatric Hematology and Oncology, Collegium Medicum, University of Zielona Góra, Zielona Góra, Poland; ^15^ Clinic of Pediatric Hematology and Oncology, Medical University of Lublin, Lublin, Poland

**Keywords:** hemophilia A, annualized bleeding rate, annualized joint bleeding rate, plasma-derived FVIII, recombinant FVIII, bleeding prophylaxis, children

## Abstract

**Introduction:**

In 2020, the new nationwide protocol of prophylaxis in Polish plasma-derived FVIII (pdFVIII) previously treated patients (PTPs) with severe hemophilia A (sHA) was introduced, resulting in the necessity of switching from pdFVIII to recombinant FVIII (octocog-alpha; rFVIII). The study aimed to: (1) assess the safety of switching from pdFVIII to rFVIII, (2) assess the safety and efficacy of pharmacokinetically based (PK-based) personalized prophylaxis in severe hemophilia A.

**Patients and methods:**

151 children and adolescents receiving prophylaxis with a standard dose (40 U/kg 3 x weekly) of pdFVIII were included in this study. Annualized bleeding rate (ABR) and annualized joint bleeding rate (AJBR) were analyzed for all patients before enrollment. Using myPKFiT application, pharmacokinetic (PK) analysis followed by the selection of the optimal model of prophylaxis was performed in all patients. Two possible models of prophylaxis (standard-dose rFVIII versus PK-based rFVIII) were discussed, with parents leaving the choice to their decision. Parents reported all episodes of bleeds. Screening for inhibitor was performed every 3 months. ABR and AJBR were prospectively analyzed again after a minimum follow-up time of 26 weeks.

**Results:**

141/151 (93.4%) patients completed the study. 34 patients decided to continue standard prophylaxis with rFVIII (Group I), whereas 107 were switched to PK-based prophylaxis (Group II). The risk of inhibitor development could be assessed in 137/151 (90.7%) patients. Only 2/137 (1.47%) patients (both on PK-based prophylaxis) developed low-titer inhibitor with its spontaneous elimination. The retrospective analysis of bleeds during the last 12 months of standard pdFVIII prophylaxis revealed that patients who decided to continue standard prophylaxis had historically lower ABR and AJBR than those who started PK-based personalized prophylaxis. After a minimum of 26 weeks, ABR and AJBR improved significantly in both groups. There was no significant difference in ABR and AJBR between Group I and Group II during the follow-up period. However, the rate of reduction of ABR and AJBR was higher in patients on PK-based personalized prophylaxis.

**Conclusion:**

(1) Switching from pdFVIII to rFVIII (octocog-alpha) in PTPs with sHA is safe, (2) PK-based personalized prophylaxis may decrease ABR and AJBR in children and adolescents with sHA.

## Introduction

Prophylaxis treatment for prevention of joint bleed is the optimal management in children with severe hemophilia where resources are available. It has been concluded by Manco-Johnson et al. that prophylaxis with recombinant factor VIII (rFVIII) can prevent joint damage and decrease the frequency of joint and other hemorrhages in young boys with severe hemophilia A (sHA) (1). However, the Joint Outcome Study showed that even though joints outcomes in hemophilia are better in young adults if prophylaxis is started before the age of 2.5 years compared with after age 6 years, standard FVIII prophylaxis is insufficient to fully protect joints from damage through adolescence in sHA (2). This observation becomes even more critical in light of the new definition of prophylaxis given by the World Federation of Hemophilia (WFH), which describes it as “the regular administration of a hemostatic agent/agents with the goal of preventing bleeding in people with hemophilia while allowing them to lead active lives and achieve the quality of life comparable to non-hemophilic individuals” (3). It indicates the urgent need to improve care methods for people with hemophilia.

Over a decade ago, Collins et al. stated that the pharmacokinetic (PK) response to factor VIII varies between patients, and this has important clinical implications for prophylactic treatment (4). The concept of tailored prophylaxis was extensively discussed by Reininger and Chehadeh (5). They suggested that age, lifestyle, bleeding phenotype, and pharmacokinetics of FVIII of every individual patient should be considered while designing a prophylaxis model. However, prophylaxis should be initiated before the first bleed, so PK remains the essential factor in selecting the optimal form of it. There is evidence based on real-world data that PK-driven prophylaxis may reduce the number of bleeds in children with sHA thus improving their quality of life (6).

In Poland, prophylaxis for children with sHA was initiated in 2008 (7). It is organized as a nationwide treatment program arranged and managed centrally by the Mother and Child Institute in Warsaw and reimbursed by the Polish healthcare provider (National Health Fund). From the onset, it was run as standard prophylaxis (25–40 U/kg of FVIII given three times weekly).

In the beginning, only plasma-derived (pd) products were used. Since 2010, rFVIII has been available only for previously untreated patients (PUPs). As a result of these strict regulations, two groups of patients coexisted until autumn 2020; children and adolescents with sHA on prophylaxis factor replacement therapy who were started on pdFVIII before 2010 and children who have been started on prophylaxis factor replacement therapy with rFVIII since 2010. However, in the autumn of 2020, as the result of the national tender, the new nationwide protocol of bleeding prophylaxis in Polish pdFVIII treated PTPs with sHA was introduced, resulting in the necessity of switching from pdFVIII to rFVIII (octocog-alpha; Advate; Takeda). This new protocol allows increasing the dose up to a maximum of 720 U/kg/28 days if indicated by the result of PK analysis performed in every individual patient. It created the opportunity to perform the nationwide multicenter study.

### Aim of the study

1. to assess the safety of switching from pdFVIII to rFVIII,
2. to assess the safety and efficacy of pharmacokinetic-tailored (PK-tailored) personalized prophylaxis in children and adolescents with sHA and
3. to assess the consumption of rFVIII in children on standard vs. PK-tailored prophylaxis.

## Patients and methods

This study was conducted between September 2020 and March 2022. Children and adolescents who were initially on a standard prophylaxis regimen with pdFVIII (Immunate; Takeda) were later switched to rFVIII, either as standard prophylaxis (40 U/kg, three times per week) or PK-tailored prophylaxis, were qualified for the study. Data on bleeding episodes during the last 12 months were extracted from patients’ e-diaries and verified by the local treatment coordinators in all 15 Polish pediatric hemophilia care centers. Based on these data, annualized bleeding rate (ABR) and annualized joint bleeding rate (AJBR) were calculated for every patient before enrollment. When the household supplies of pdFVIII expired, every patient continued mentioned above standard prophylaxis with rFVIII (octocog-alpha; Advate; Takeda). Using myPKFiT application, pharmacokinetic (PK) analysis followed by the selection of optimal (dosing and timing) prophylaxis model was performed in all patients as soon as possible, not later than within first ten exposure days. The highest possible trough level (recommended minimum 1%–3%) was a target for adjustment however limited by the maximum dose (not to exceed 720 U/kg/28 days) as well as the FVIII vial content (it was assumed not to waste any extra product left in the vial over the calculated dose, e.g., if the estimated dose was 1,100 U, the real-world dose was 1,000 U because the smallest vial contains 250 U so 150 U would be wasted). All pros and cons of the two possible models of prophylaxis (standard-dose rFVIII versus PK-tailored rFVIII) were discussed with parents/guardians and patients leaving the decision to them. Episodes of bleeds in two study groups were reported by parents/guardians using e-diaries and verified by the local hemophilia treaters. Screening for inhibitor development was performed every 3 months and in every case of clinical suspicion when clinically indicated. ABR and AJBR were analyzed again after a minimum follow-up time of 26 weeks concerning models of prophylaxis (standard vs. PK-tailored) and a type of FVIII (pdFVIII vs. rFVIII) used. Values of ABR and AJBR in respective groups of patients were compared using the Student's *t*-test and the Wilcoxon signed-rank test.

The study was reviewed and approved by Bioethics Committee in Pomeranian Medical University, Szczecin, Poland. Written informed consent to participate in this study was provided by the participants’ legal guardian/next of kin.

## Results

A total of one hundred fifty-one PTPs (all boys) aged 93–209 (median 166) months receiving prophylaxis previously with a standard dose (40 U/kg 3 x weekly) of pdFVIII (Immunate; Takeda) were included in this study of which, due to COVID-19 pandemic-related problems, 141/151 (93.4%) patients aged 94–209 months (mean 167, median 178, IQR 156.5–203.7 months) completed the study. None of patients entering the study had an inhibitor.

Thirty-four patients decided to continue standard prophylaxis with rFVIII (Group I), whereas one hundred-seven started PK-tailored prophylaxis (Group II) with the same factor. The age distribution of studied patients is presented in Table 1.

**Table 1:** The age distribution of studied patients.

	0–6 years	>6–12 years	>12–18 years	Total number
Group I (Standard prophylaxis)	0	4	30	34
Group II (PK-tailored prophylaxis)	0	14	93	107

The risk of inhibitor development could be assessed in 137/151 (90.7%) patients. Only 2/137 (1.47%) patients (both on PK-tailored prophylaxis) developed low-titer inhibitor (0.8 BU/ml after 120 EDs and 1.8 BU/ml after 78 EDs, respectively) with no clinical manifestation and with spontaneous elimination of inhibitor in both cases in which the management remained unchanged.

The retrospective analysis of bleeds during the last 12 months of standard pdFVIII prophylaxis revealed that patients who decided to continue standard prophylaxis (Group I) had, before switching the factor product to rFVIII, lower ABR and AJBR than those who started PK-based personalized prophylaxis (Group II) with rFVIII (mean ABR 1.09 vs. 2.06; *p* = 0.04; mean AJBR 0.41 vs. 0.78 *p* = 0.058 respectively). The consumption of pdFVIII was comparable in both groups (Group I mean 404.4 U/kg/28 days vs. Group II mean 403.2 U/kg/28 days; *p* > 0.05). These results are presented in Table 2.

**Table 2:** Baseline patient characteristic prior to switching from pdFVIII to rFVIII.

	Group I standard pdFVIII prophylaxis (*n* = 34)	Group II standard pdFVIII prophylaxis (*n* = 107)	*p* value
Age of patients (mo.)	Range: 94–155; mean: 137.7 ± 14.4; median: 144.5 (IQR 127.5–144.5)	Range: 94–206; mean 175.6 ± 31.1; median: 175.0 (IQR 159.0–196.0)	*p* = 0.002
ABR	Range: 0–8; median: 0; mean: 1.09 ± 1.74	Range: 0–43; median: 0; mean: 2.06 ± 5.0	*p* = 0.04
AJBR	Range: 0–4; median: 0; mean: 0.41 ± 0.96	Range: 0–12; median: 0; mean: 0.78 ± 1.71	*p* = 0.058
pdFVIII single dose (U/kg)	Range: 21.9–40.4; median: 33.1; mean: 33.7 ± 5.65	Range: 21.5–48.4; median: 34.0; mean: 33.6 ± 6.32	*p* = NS
pdFVIII consumption (U/kg/28 days)	Range: 262.8–484.8; median: 393.2; mean: 404.4 ± 91.79	Range: 258.0–580.0; median: 244.7; mean: 403.2 ± 82.89	*p* = NS

After a minimum of 26 weeks from enrollment, ABR and AJBR improved significantly in both groups (Group I standard pdFVIII vs. standard rFVIII prophylaxis: mean ABR 1.09 vs. 0.44; *p* = 0.02; mean AJBR 0.41 vs. 0.17; *p* = 0.03 respectively; Group II standard pdFVIII vs. PK-tailored rFVIII prophylaxis: mean ABR 2.06 vs. 0.63; *p* = 0.001; mean AJBR 0.78 vs. 0.27; *p* = 0.001 respectively). These results are presented in Tables 3, 4. There was no significant difference in ABR and AJBR between Group I and Group II (mean ABR 0.44 vs. 0.63; *p* > 0.05, mean AJBR 0.17 vs. 0.27; *p* > 0.05), during the follow-up period. However, the rate of reduction of ABR and AJBR was higher in patients on PK-tailored personalized prophylaxis (Group I vs. Group II: ABR reduction rate 56.6% vs. 69.4%; AJBR reduction rate 58.5% vs. 65.4%). The consumption of rFVIII was higher in patients on PK-tailored prophylaxis (Group I mean 443.4 U/kg/28 days vs. Group II mean 482.4 U/kg/28 days; *p* = 0.03). These data are presented in Table 5. Among Group II (PK-tailored prophylaxis), the frequency of dosing increased (every 2nd day) in 84 pts (78.5%), remained unchanged (3 times per week) in 22 pts (20.5%) and decreased (2 times per week) in 1/107 pt. (0.9%). The dose of each infusion (IU/kg) decreased in 40 pts (37.4%), remained unchanged in 5 pts (4.7%), and increased in 62/107 pts (57.9%).

**Table 3:** Comparison of bleeding outcomes in patients on standard prophylaxis regimen, before and after FVIII product switch.

	Group I standard pdFVIII prophylaxis (*n* = 34)	Group I standard rFVIII prophylaxis (*n* = 34)	*p* value
ABR	Range: 0–8; median: 0; mean: 1.09 ± 1.74	Range: 0–2.99; median: 0; mean: 0.44 ± 0.78	*p* = 0.02
AJBR	Range: 0–4; median: 0; mean: 0.41 ± 0.96	Range: 0–1.79; median: 0; mean: 0.17 ± 0.45	*p* = 0.03

**Table 4:** Comparison of bleeding outcomes in patients on PK-tailored prophylaxis regimen, before and after FVIII product switch.

	Group II standard pdFVIII prophylaxis (*n* = 107)	Group II PK-tailored rFVIII prophylaxis (*n* = 107)	*p* value
ABR	Range: 0–43; median: 0; mean: 2.06 ± 5.0	Range: 0–8.8; median: 0; mean: 0.63 ± 1.32	*p* = 0.001
AJBR	Range: 0–12; median: 0; mean: 0.78 ± 1.71	Range: 0–4.35; median: 0; mean: 0.27 ± 0.73	*p* = 0.001

**Table 5:** Comparison of bleeding rates and factor consumption in patients on standard prophylaxis group vs. PK-tailored prophylaxis group.

	Group I standard rFVIII prophylaxis (*n* = 34)	Group II PK-tailored rFVIII prophylaxis (*n* = 107)	*p* value
ABR	Range: 0–2.99; median: 0; mean: 0.44 ± 0.78	Range: 0–8.8; median: 0; mean: 0.63 ± 1.32	*p* = NS
AJBR	Range: 0–1.79; median:0; mean: 0.17 ± 0.45	Range: 0–4.35; median: 0; mean: 0.27 ± 0.73	*p* = NS
rFVIII single dose (U/kg)	Range: 26.1–54.7; median: 34.8; mean: 35.4 ± 5.67	Range: 21.1–56.5; median: 34.6; mean: 35.3 ± 6.32	*p* = NS
rFVIII consumption (U/kg/28 days)	Range: 313.0–765.8; median: 420.2; mean: 443.4 ± 91.79	Range: 289.2–678.0; median: 479.9; mean: 482.4 ± 82.89	*p* = 0.03

**Figure 1:**
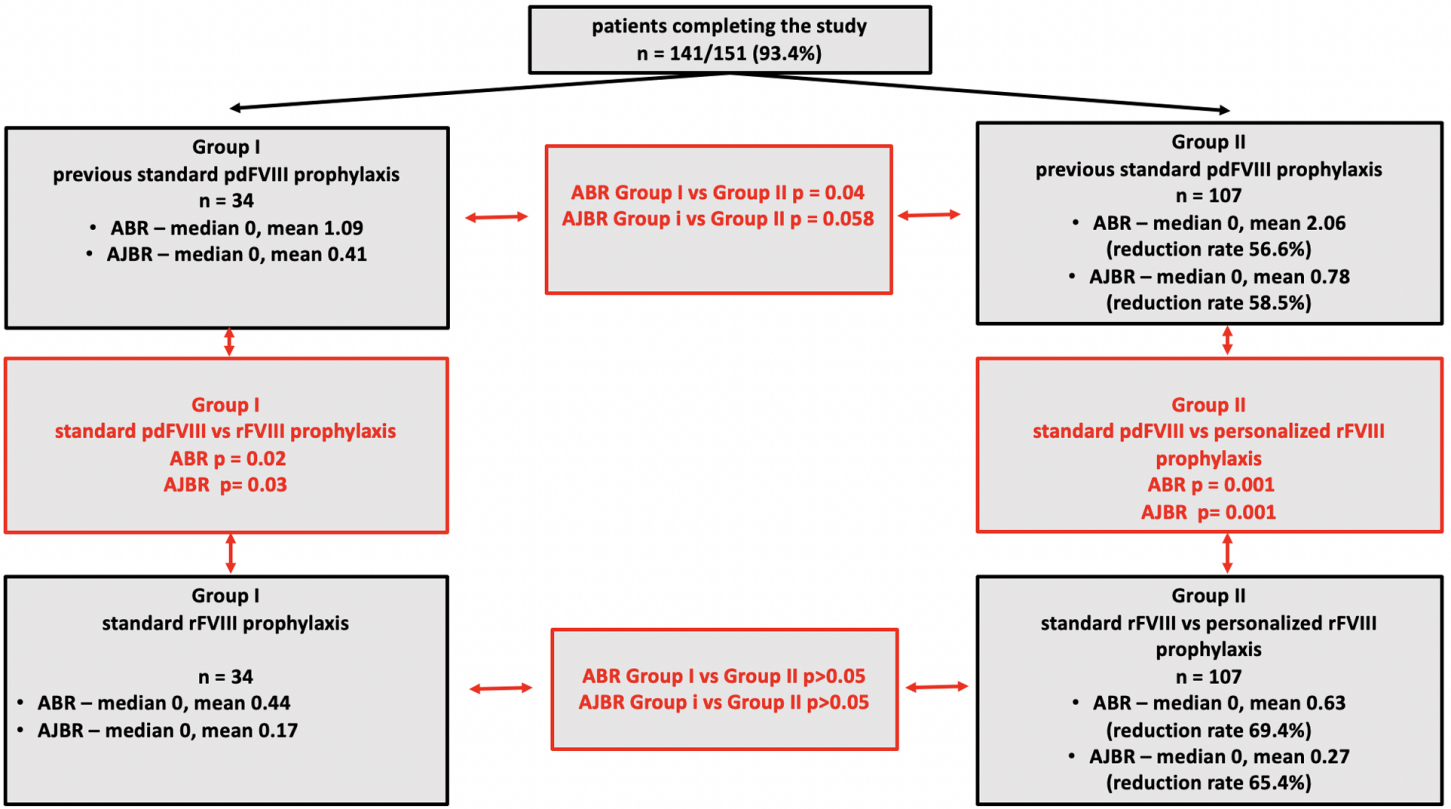
The graphic summary of the study and its results.

The summary of the study and its results is presented in graphic form in Figure 1.

## Discussion

Even though there is real progress in the field of non-factor drugs and gene therapy, factor VIII concentrate administration, regardless of its origin, remains in many countries—for children and adolescents with sHA—the therapy, which is at least temporarily able to restore normal hemostasis (8). Since plasma resources are not unlimited and rFVIII concentrates are getting cheaper (sometimes even more affordable than pd preparations), we will still face the need to switch our patients from plasma-derived to recombinant factors. For many years such a maneuver was associated with the fear of inhibitor development. It is commonly believed now that switching is safe. It has been stated by Santagostino et al. that current evidence does not suggest that switching products significantly influences inhibitor development (9). This conclusion is based mainly on the results of the three largest “switching” studies from Ireland, Canada, and the UK; however, populations observed and analyzed by the authors were formed by both children and adults (10–12). Some recent studies, like the one based on real-world data from the TAURUS study, also indicate that switching from different FVIII products to octocog-alpha is safe with respect to inhibitor development since no one from 160 patients developed inhibitor however, all of them were also adults (13). Data on inhibitor development related to factor switching in children are still scarce. In a recent publication, Huang et al. reported that none of 47 boys aged less than 18 years switched to one of three standard half-life factors (Kovaltry, Advate, and GreeMono) developed inhibitor (14). Similar results were published by Escuriola-Ettinghausen et al. however, only 14 out of 68 patients switched to turoctokog-alpha were younger than 12 years (15). To our best knowledge, we present data on the safety of switching in one of the largest (137 patients) pediatric populations ever studied. Our data indicate that switching from plasma-derived (Immunate; Takeda) to recombinant (Advate; Takeda) factor VIII concentrate in children is safe. Only 2 of 137 patients developed low titre inhibitor with no clinical manifestation and spontaneous resolution on unchanged prophylaxis. None of these patients had history of previous inhibitor or family history of inhibitors. Types of mutations in these patients were not studied.

Analysis of the results of standard pdFVIII prophylaxis in both studied groups (I and II) revealed that they are still unsatisfactory. Even though at least 50% of patients did not experience any bleeding episodes (median ABR and ABJR—0 in both groups), a large group of patients suffered from it despite prophylaxis. It prompted us to change the concept of prophylaxis. Recent guidelines and recommendations suggest that prophylaxis should be adjusted to the individual requirements of every patient and that one of the most important factors which should be taken under consideration is the result of PK analysis of the factor used for prophylactic treatment (3, 16).

As the result of the national tender in autumn 2020, octocog-alpha (Advate; Takeda) was selected for the continuation of prophylaxis in all Polish PTPs; the “by-product” of this contract was the access to myPKFiT application explicitly designed not only to study pharmacokinetics of octocog-alpha but also to perform simulations of different models of prophylaxis to optimize it for every individual patient. This application is based on the Bayesian model allowing to perform PK analysis using only two samples of blood, thus sparing children's blood, and was previously used by other groups. Using this tool, Mingot-Castellano et al. adjusted prophylactic regimens to PK parameters, resulting in a significant reduction of ABR and AJBR with no significant increase in factor VIII consumption in the majority of 37 patients (17). Similar results were reported by Megías-Vericat et al. (18). Results presented in this study indicate that PK-tailored prophylaxis may improve the clinical outcome in children with sHA. Both: ABR, as well as AJBR, were significantly lower in Group I and Group II on rFVIII prophylaxis; however, the most significant improvement was observed in Group II. The introduction of PK-tailored prophylaxis eliminated the significant differences in ABR and AJBR observed between patients of Groups I and II on standard pdFVIII prophylaxis. ABR and AJBR in Group I and Group II on rFVIII prophylaxis did not differ significantly (see Figure 1).

There is growing evidence that PK-tailored prophylaxis may improve the clinical outcome in children with sHA. In the recently published study, Huang et al. reported that in pediatric patients, personalized and based on individualized target trough level (which is also a PK-related parameter), bleeding prophylaxis protocols are effective in reducing bleeds (6). Other studies also support this statement (19).

Not surprisingly, the mean rFVIII consumption (U/kg/28 days) was higher in patients receiving PK-tailored rFVIII prophylaxis. This is probably due to the fact that the t1/2 of recombinant FVIII is relatively short, so maintaining safe trough levels requires more frequent dosing (3 times a week in group I patients on standard prophylaxis vs. every other day in group II patients on PK-adapted prophylaxis. In addition, mean rFVIII consumption was only 8.7% higher in patients on PK-tailored prophylaxis, and none exceeded the maximum dose allowed by the protocol. Mingot-Castellano et al. and Megias-Vericat et al. claimed that using the myPKFiT app for PK analysis allows treatment optimization (17, 18). Our data suggest the same: it is possible to achieve better outcomes with a relatively small increase in factor usage. It may be important because economic factors continue to play a decisive role even in welfare countries; hemophilia treatment remains one of the most expensive procedures among all medical interventions, and we all need to do everything we can to optimize this expenditure while maintaining the primary goal of reducing bleeding in our patients (20).

Finally, two major limitations of this study should be mentioned. First: rates of bleeds were surprisingly low. The study was conducted during the period of COVID-19 pandemic, so COVID-19 restrictions might have an impact on the physical activity of children, which are most active staying together at school or kindergarten however, this factor inhibited the risk of traumatic bleeds in both Groups (I and II) of patients thus not influencing the difference in ABR and AJBR. Second: the study is based on real-world data, thus posing the question regarding their reliability. Parents or guardians of children with hemophilia tend to overestimate the rate of bleeds because all hemophilia treaters train them to respect the principle “factor first”. However, it also refers to parents or guardians of children in both studied groups, so it should not impact this study's results.

## Conclusion

We believe that the results presented in this study allow us to form the following conclusions:

1. Switching from pdFVIII to rFVIII (octocog-alpha) in PTPs with sHA is safe.
2. PK-tailored personalized prophylaxis may decrease ABR and AJBR in children and adolescents with sHA, especially in those whose results of previous prophylaxis were unsatisfactory.
3. While improving the clinical outcome, PK-tailored prophylaxis may optimize the consumption of octocog-alpha in children with sHA.

## Data Availability

The raw data supporting the conclusions of this article will be made available by the authors, without undue reservation.
